# The cyclic nucleotide 3’,5’-cIMP produced by sGC is a second messenger in the vascular wall

**DOI:** 10.1186/2050-6511-16-S1-A25

**Published:** 2015-09-02

**Authors:** Paul M Vanhoutte, Susan WS Leung, Yuansheng Gao

**Affiliations:** 1 Department of Pharmacology & Pharmacy and State Key Laboratory for Pharmaceutical Biotechnology, Li Ka Shing Faculty of Medicine, the University of Hong Kong, Hong Kong S.A.R., China; 2 Department of Physiology and Pathophysiology, Peking University Health Science Centre, Beijing, China

## 

Traditionally, only the 3’,5’-cyclic monophosphates of adenosine and guanosine (produced by adenylyl cyclase and guanylyl cyclases, respectively) are regarded as true ‘second messengers’ in the vascular wall, despite the presence of other cyclic nucleotides in different tissues. Among these non-canonical cyclic nucleotides, inosine 3’,5’-cyclic monophosphate (cIMP) is synthesized by soluble guanylyl cyclase in porcine coronary arteries in response to hypoxia, when the enzyme is activated by endothelium-derived nitric oxide. Its production is associated with augmentation of vascular contraction mediated by stimulation of Rho kinase. Similar endothelium-dependent, NO-dependent and soluble guanylyl cyclase-dependent contractions can be evoked with thymoquinone, which also augments the levels of cIMP. Based on these findings, cIMP appears to meet most, if not all, of the criteria required for it to be accepted as a ‘second messenger’, at least in the vascular wall. The understanding of the role of this non-canonical cyclic nucleotide may help identifying novel therapeutic targets for certain cardiovascular disorders, in particular those associated with sleep apnea.

